# Penile strangulation, a study of two cases

**DOI:** 10.1016/j.ijscr.2022.107100

**Published:** 2022-04-21

**Authors:** Ganesh Bhakta Acharya, Suman Baral, Abishek Poudel, Ananda Neupane, Kamal Kandel

**Affiliations:** a Department of Urology, Manipal College of Medical Sciences, Pokhara, Nepal; b Department of Surgery, Dirghayu Pokhara Hospital, Pokhara, Nepal; c Department of Surgery, Manipal College of Medical Sciences, Pokhara, Nepal; d Kathmandu University, Nepal

**Keywords:** Jumbo cutter, Metallic ring, Penile strangulation

## Abstract

**Introduction:**

Penile strangulation is one of the rarest clinical findings. So, we aimed to present the clinical case of two cases that we encountered at our institute.

**Presentation of cases:**

Case 1. A 26-year-male with bipolar disorder presented to emergency room with complaints of metallic ring entrapment in the penis for 4 days. A gold-plated metallic ring measuring 1 × 0.25 cm was found encircling the coronal sulcus along with foul-smelling pus and slough underneath as a result of necrosis of the skin. Jumbo cutter was used to cut the ring. Loose sutures were applied to the injured part with debridement. Case 2. A 66-year-old man presented to emergency with complaints of penile swelling and poor urinary stream for the last 2 days. Local examination showed a plastic ring of water bottle seen stuck at the root of penis. Distal penis was edematous with multiple areas of skin discoloration and petechia. The ring was cut with simple surgical scissor.

**Discussion:**

This clinically emergency condition may lead to wide range of vascular and mechanical injuries if the treatment is delayed. Plastic rings can be easily cut out whilst metallic objects which are thick and hard are difficult to remove. Pliers' application or use of jumbo cutter as in one of our cases is beneficial for prompt release of edematous pressure and salvage of the penis.

**Conclusion:**

Penile strangulation due to foreign body is a rare clinically emergency condition. Urgent intervention is necessary to remove the object causing constriction along the penis.

## Highlights

• Penile strangulation due to foreign body entrapment is one of the rarer emergency conditions in urology.
• This may lead to wide range of vascular and mechanical injuries if the treatment is delayed.
• Urgent intervention is necessary to remove the object causing constriction along the penis.

## 1 Introduction

Penile strangulation is one of the uncommon presentations in urology and even strangulation by the foreign body is rare. Mostly, this clinical condition is associated with an attempt of the individual for persistent arousal of penis for sexual gratification using the rings through the penis [1]. As evidenced in the literatures, commonly used apparatus included heavy metal rings, hammer head, plastic bottle necks, sprockets, and plumbing cuffs [2]. Time factor plays a critical role in the management of such clinical condition as the late presentation or intervention might deem penis to strangulate leading to gangrene and amputation [3]. Timely decompression saves the penis and the resources availability like jumbo metal cutter and help from colleagues may need to be accessed as soon as possible when urologists and surgeons may not be aware about the use. So, we present the clinical cases of two patients that we encountered at our institute where a metallic ring and bottle neck ring were used. The patients had good clinical outcome due to timely intervention. The work has been reported in line with the SCARE 2020 criteria [4].

## 2 Case report

### 2.1 Case 1

**Fig. 1:**
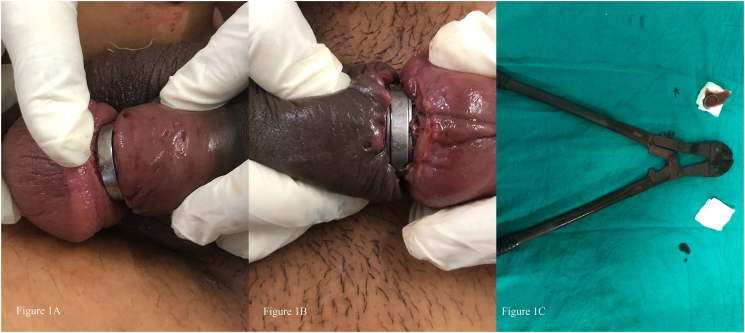
Figure 1A shows the metallic ring encircling the coronal sulcus at dorsal surface Figure 1 B shows the ventral surface of the penis with sloughed out tissue underneath the metallic ring associated with foul smelling discharge Figure 1C shows the orthopedics jumbo cutter along with cut off metallic ring

A 26 years male patient with the history of bipolar disorder under medication with mood stabilizers and anti-psychotics presented to emergency room with complaints of metallic ring entrapment in the penis for 4 days. The patient developed gradual progressive swelling of the glans of penis distal to the ring site. Following the incident, the patient tried to remove the ring, however remained unsuccessful. There was no history of intoxication or drug abuse. On local examination, a gold-plated metallic ring measuring 1 × 0.25 cm was found encircling the coronal sulcus. Gross edema and congestion of the glans penis and prepuce distal to the metal ring was seen along with foul-smelling pus and slough underneath the metal ring as a result of necrosis of the skin due to the strangulating effect (Fig. 1A and B). There was no blackish discoloration or signs of necrosis of distal penile shaft with no evidence of any urethro-cutaneous fistula. Urinary stream was poor but the patient could pass the urine. Under spinal anesthesia, multiple attempts to remove the ring by application of xylocaine jelly, needle prick to the edematous part to decompress, use of Gigli saw wire to cut off the ring failed. Finally, orthopedics jumbo cutter was used to cut the ring (Fig. 1C). There was no deep injury to the underlying structures. Loose sutures were applied to the injured part with careful debridement. Circumcision was avoided. Psychiatric consultation was done and the patient got discharged on second post operative day. Follow up examination after a month showed no evidence of urinary or sexual problems.

### 2.2 Case 2

A 66-year-old man, chronic alcoholic presented to emergency with complaints of penile swelling and poor urinary stream for the last 2 days. Patient gave history of insertion of plastic ring on the penis for the sexual gratification under the influence of alcohol. There was no history of trauma.

**Fig. 2:**
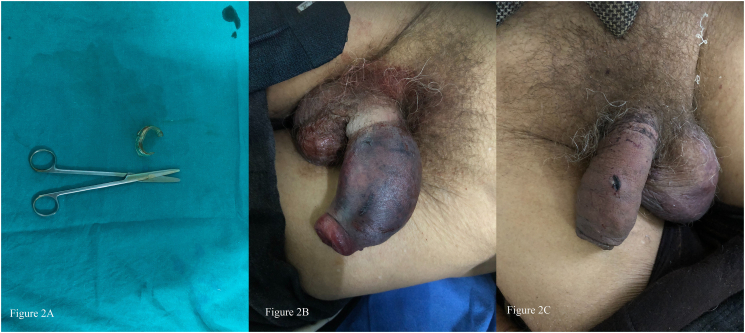
Figure 2A shows the surgical scissor along with cut out plastic bottle ring Figure 2B shows the edematous distal part with impingement at root of penis along with multiple areas of skin discoloration and petechia Figure 2C shows the recovering penis with decreasing edema at 7th day follow up

During presentation, the signs of alcohol intoxication were not evident. Vitals were stable. Local examination showed a plastic ring of water bottle seen stuck at the root of penis. Distal penis was edematous with multiple areas of skin discoloration and petechia (Fig. 2B). The ring was cut with simple surgical scissor (Fig. 2A). Circumcision was avoided. The patient was admitted to observe for complications. The patient got discharged after two days with no complications. Follow up examination after 7 days showed decreased swelling and edema with normal contour of the penis (Fig. 2C). One month follow up examination showed no evidence of urinary or sexual problems.

## 3 Discussion

The cases of penile strangulation have been reported worldwide which is rare. Till date, around 60 cases have been reported in the literatures since Gauthier reported the first case in 1755 [5], [6]. This clinically emergency condition may lead to wide range of vascular and mechanical injuries if the treatment is delayed, characterized by the loss of penile sensation, ischemic skin necrosis, urethral injury, urethra-cutaneous fistula and even penile amputation [7].

Bhat et al. in 1995 proposed a classification of penile incarceration which composed of 5 grades ranging from edema to necrosis as follows [8] (Table 1).

**Table 1:** Grades of penile strangulation ranging from edema to necrosis.

Grade	Description
1	Edema of the distal penis
2	Injury to penile skin constriction of corpus spongiosum without any urethral injury. Edema of distal penis with decrease sensation
3	Injury to skin and urethra but no urethral fistula. Loss of distal penile sensation
4	Complete division of corpus spongiosum leading to urethral fistula and constriction of corpus cavernosa with loss of distal penile sensation
5	Gangrene, necrosis or complete amputation of the distal penis

As per the classification, both of our cases fell upon Grade 2.

Early intervention is deemed necessary to have the favorable outcome. Treatment depends upon the degree and material of entrapment along with distal edema associated with it. Nonmetallic objects like plastic rings can be easily cut out as one of our cases whilst metallic objects which are thick and hard are difficult to remove with chisel, saw or cutter [5]. Various reported techniques have been published in literatures [5], [7], [9]. An engorged penis makes it difficult for the constricting object to come out easily, so a string or wire is inserted through the object and the penile skin along with gauze so that the object slips off easily along with aspiration of corporal blood. For thick objects, application of common surgical instruments may not be warranted. Use of chisel, saw or cutter may be unsuccessful. Pliers' application or use of jumbo cutter as in one of our cases is beneficial for prompt release of edematous pressure and salvage of the penis [1]. Application of metal cutter produces heat within the metal which should be cooled during the procedure. The small room between metal and penile skin should be dealt with caution in order to remove chances of injury to penis during intervention. Cold normal saline can be used to sprinkle the penile tissue along with the cutter blade [1]. Skin ulcerations and necrosis requires careful debridement with primary closure of the defect whilst severe cases might require surgical repair of urethra-cutaneous fistula [5]. Autoamputation may be the utmost sequelae of this clinical condition [1].

## 4 Conclusion

Penile strangulation due to foreign body is a rare clinically emergency condition. Urgent intervention is necessary to remove the object causing constriction along the penis. Availability of pliers and removal tools should be assessed as soon as possible for quick removal and salvage of the penis.

## Provenance and peer review

Not commissioned externally peer reviewed.

## Informed consent

Written informed consent was obtained from the patient for publication of this case report and accompanying images. A copy of the written consent is available for review by the Editor-in-Chief of this journal on request.

## Ethical approval

Ethical approval was not mandatory for publication of case reports as per the institutional policy.

## Funding

This case report did not receive any specific grant from funding agencies in the public, commercial, or not-for-profit sectors.

## Guarantor

Suman Baral.

## Research registration number

1. Name of the registry: NA
2. Unique identifying number or registration ID: NA
3. Hyperlink to your specific registration (must be publicly accessible and will be checked): NA.

## CRediT authorship contribution statement

Design and Idea: Suman Baral, Ganesh Bhakta Acharya, Abishek Poudel
Drafting: Suman Baral, Ganesh Bhakta Acharya, Abishek Poudel
Final Revision: Suman Baral, Ganesh Bhakta Acharya, Abishek Poudel, Ananda Neupane, Kamal Kadel.

## Declaration of competing interest

Authors declare that there are no any conflicts of interest regarding publication of the manuscript.
